# Naive infection predicts reservoir diversity and is a formidable hurdle to HIV eradication

**DOI:** 10.1172/jci.insight.150794

**Published:** 2021-08-23

**Authors:** Marilia R. Pinzone, Sam Weissman, Alexander O. Pasternak, Ryan Zurakowski, Stephen Migueles, Una O’Doherty

**Affiliations:** 1 Department of Pathology and Laboratory Medicine, University of Pennsylvania, Philadelphia, Pennsylvania, USA.; 2 Department of Medical Microbiology, Amsterdam UMC, University of Amsterdam, Laboratory of Experimental Virology, Amsterdam, Netherlands.; 3 Department of Biomedical Engineering, University of Delaware, Newark, Delaware, USA.; 4 HIV-Specific Immunity Section of the Laboratory of Immunoregulation, National Institute of Allergy and Infectious Diseases, NIH, Bethesda, Maryland, USA.

**Keywords:** AIDS/HIV, Infectious disease, T cells

## Abstract

Historically, naive cells have been considered inconsequential to HIV persistence. Here, we compared the contributions of naive and memory cells to the reservoirs of individuals with a spectrum of reservoir sizes and variable immunological control. We performed proviral sequencing of approximately 6000 proviruses from cellular subsets of 5 elite controllers (ECs) off antiretroviral therapy (ART) and 5 chronic progressors (CPs) on ART. The levels of naive infection were barely detectable in ECs and approximately 300-fold lower compared with those in CPs. Moreover, the ratio of infected naive to memory cells was significantly lower in ECs. Overall, the naive infection level increased as reservoir size increased, such that naive cells were a major contributor to the intact reservoir of CPs, whose reservoirs were generally very diverse. In contrast, the reservoirs of ECs were dominated by proviral clones. Critically, the fraction of proviral clones increased with cell differentiation, with naive infection predicting reservoir diversity. Longitudinal sequencing revealed that the reservoir of ECs was less dynamic compared with that of CPs. Naive cells play a critical role in HIV persistence. Their infection level predicts reservoir size and diversity. Moreover, the diminishing diversity of the reservoir as cellular subsets mature suggests that naive T cells repopulate the memory compartment and that direct infection of naive T cells occurs in vivo.

## Introduction

The persistence of a reservoir of latently infected resting cells underlies the need for lifelong antiretroviral therapy (ART) in individuals with HIV infection, because ART interruption usually results in rapid viral rebound and eventual CD4 T^+^ cell depletion (1). Memory CD4^+^ T cells have been considered the major HIV reservoir for multiple reasons. First, activated T cells are more susceptible to HIV so it logically follows that the reservoir formed when activated T cells returned to a resting memory state (2, 3). Consistent with this hypothesis, memory cells were shown to contain higher levels of HIV DNA than naive T cells in HIV-infected individuals (2, 4). Finally, although memory cells are heterogenous, they express higher levels of CCR5 coreceptor than naive T cells (5, 6). This last point is critically important because HIV transmission is driven by CCR5-tropic HIV (7).

Thus, naive cells have been considered inconsequential to HIV persistence. Recent work challenged this dogma, showing that naive cells were a major contributor to the intact reservoir (8–10). The proviral sequences from naive cells were distinct from memory cells, bolstering the evidence that infection was not due to contamination; however, the significance was uncertain due to the small number of participants studied. Herein, we expand our cohort to include a spectrum of reservoir sizes and immune control. We sorted CD4^+^ T cell subsets from 5 elite controllers (ECs) and 5 chronic progressors (CPs) and performed near-full-length (NFL) proviral sequencing. We demonstrated that infected naive cells contained the most diverse HIV sequences and contributed more significantly to the reservoir as its size increased. The high diversity of proviral sequences in naive cells suggests direct infection events must have occurred in vivo in CPs. On the other hand, we discovered that ECs have minimal naive infection and minimal viral diversity. These findings would imply that naive infection predicts overall reservoir size and diversity and that naive cells play a role in repopulating the memory reservoir.

## Results

### The naive reservoir was exceedingly small in ECs yet naive infection predicted overall reservoir size.

**Figure 1:**
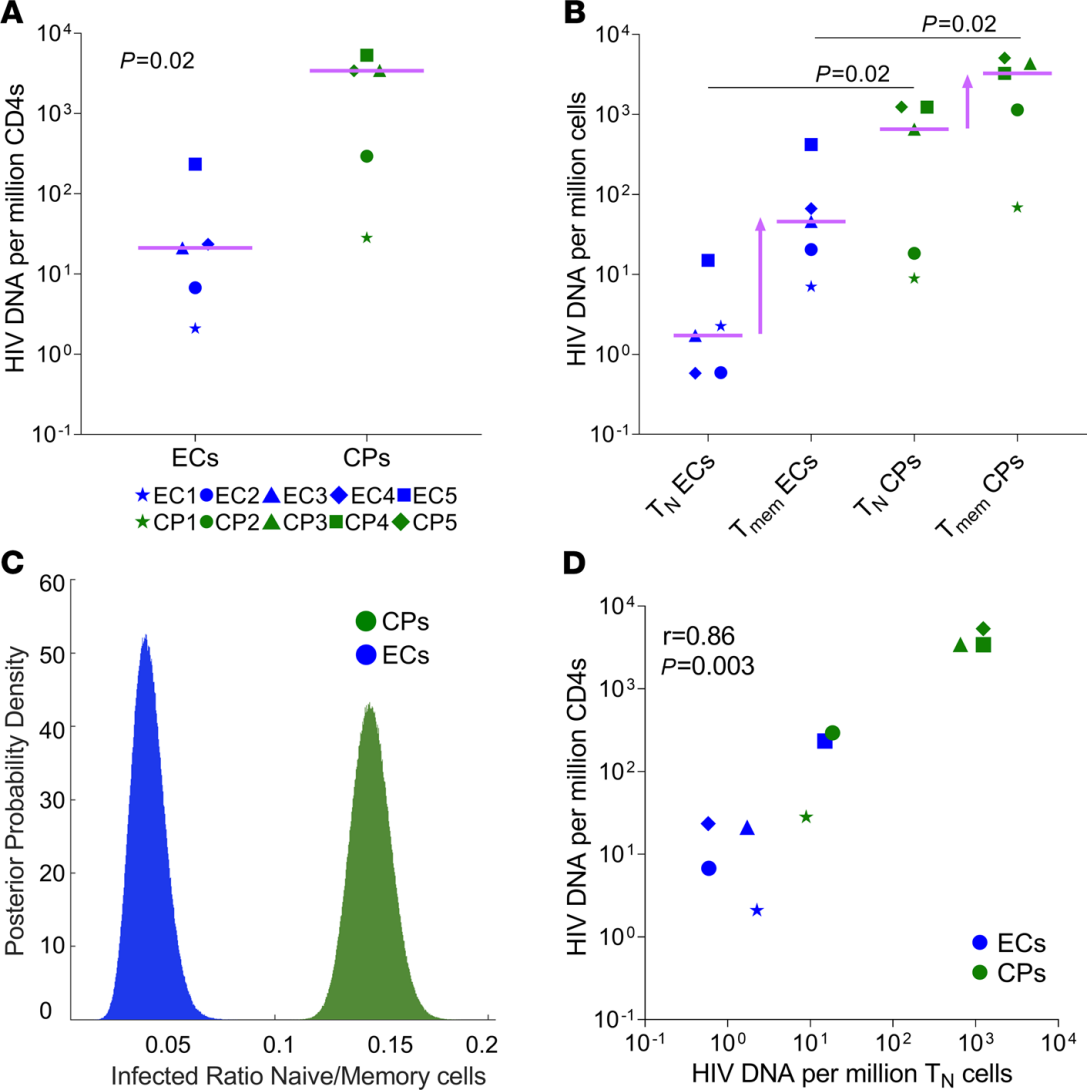
Comparison of naive and memory infection levels in ECs and CPs. (**A**) Levels of HIV DNA at enrollment in ECs (*n =* 5, blue) and CPs (*n =* 5, green). Levels of HIV DNA/million CD4^+^ T cells were significantly lower in ECs vs. CPs (21 [IQR, 4.5–129] vs. 3416 [IQR, 162–4383] copies/million CD4^+^ T cells, *P =* 0.02). (**B**) HIV DNA levels in the naive cells were approximately 300-fold lower in ECs (1.7 [IQR, 0.6–8.6] copies/million cells) compared with CPs (655 [IQR, 13.6–1239] copies/million cells, *P =* 0.02), whereas HIV DNA levels in memory cells were approximately 60-fold lower in ECs (46 [IQR, 14–244] copies/million cells) compared with CPs (3264 [IQR, 605–4718] copies/million cells, *P =* 0.02). (**C**) The ratio of naive/memory infection was significantly lower in ECs (0.04 [95% CI, 0.03–0.06]) compared with CPs (0.14 [95% CI, 0.13–0.16], *P <* 10^–6^). (**D**) Correlation between HIV DNA in naive cells and total HIV DNA (*r* = 0.86, *P* = 0.003) in ECs (*n =* 5) and CPs (*n =* 5). Lines represent median values. Groups were compared using the Mann-Whitney *U* test. Levels of HIV DNA are reported as copies/million cells. (**B**) Levels of memory infection were estimated based on levels of HIV DNA in Tcm, Tem, and Ttm cells corrected for the contribution of each subset to the CD4 population. The following formula was used: HIV DNA Tmem cells = (HIV DNA Tcm × Tcm /Total CD4) + (HIV DNA Ttm × Ttm /Total CD4) + (HIV DNA Tem × Tem /Total CD4). HIV DNA levels were estimated by qPCR with primers binding to the HIV LTR for CP2, CP3, CP4, CP5, and EC5 and by limiting dilution PCR for the remaining individuals, as described in Methods. Correlations were calculated using Spearman’s coefficient correlation. A modified Fisher’s exact test with a correction for uneven sampling of subsets was used to compare the ratio of infected naive to infected memory cells in ECs vs. CPs. ART, antiretroviral therapy; CPs, chronic progressors; ECs, elite controllers; LTR, long terminal region; naive T cells, naive CD4^+^ T cells; Tmem, memory CD4^+^ T cells.

We previously showed that naive cells significantly contribute to the HIV reservoir in 2 individuals on ART (9, 10). To expand our cohort to include individuals with a range of reservoir size and immune control, we enrolled 5 ECs and 5 CPs (Table 1, Table 2, and Supplemental Table 1; supplemental material available online with this article; https://doi.org/10.1172/jci.insight.150794DS1). For CPs, we deliberately chose individuals with a large range of reservoir sizes. As previously reported (11–13), the levels of HIV DNA were lower in ECs compared with CPs (Figure 1A; *P =* 02). Notably, ECs had predominantly CCR5-tropic proviruses, whereas only 2 of the CPs had predominant CCR5-tropic proviruses (Table 1). This is consistent with the idea that ECs experience less viral evolution over time, compared with CPs before ART initiation, with less reservoir expansion over time (14–17). Moreover, those CPs with predominant CCR5-tropic HIV infection either had a history of modest viral control before ART initiation (CP1) or were placed on ART relatively early compared with the other participants (CP2; Table 1).

To study the level of naive infection in this cohort, we sorted CD3^+^CD8^–^ T cells by flow cytometry into naive T cells, central memory T (Tcm) cells, transitional memory T (Ttm) cells, cells, and effector memory T (Tem) cells (see Methods; ref. 10). Naive cells were selected as CD45RA^+^CCR7^+^CD27^+^CD95^–^ cells to exclude stem memory T (Tscm) cells. Strikingly, most ECs had barely detectable naive infection (≤3 infected cells per million sorted naive T cells; Figure 1B). To capture this difference, we compared the ratio of infected naive cells to memory cells using a modified Fisher’s exact test, with a Monte Carlo correction for uneven sampling of subsets (see Methods). We showed significantly lower naive relative to memory infection in ECs compared with CPs (*P <* 10^–6^; Figure 1C). Thus, naive T cells are less infected in ECs compared with CPs.

Given the observation of lower levels of naive infection within ECs, we wanted to know if the levels of naive infection predict the reservoir size. Naive infection correlated with the size of the reservoir (Figure 1D; *r* = 0.86, *P =* 0.003) better than any other memory subset (*r*^2^ = 0.92, *P =* 0.00001 by linear regression; Table 3). This held true even when analyzing ECs (*r*^2^ = 0.97, *P =* 0.002) and CPs (*r*^2^ = 0.86, *P =* 0.02) separately. Given the small naive reservoir in some of these individuals, we were initially surprised by the strength of this correlation. We then recognized that the slope of linear regression for naive cells was steeper compared with memory cells and significantly different (slope 3.6 vs. 0.88, respectively, *P =* 0.002). Although we expect that the infected rate of any cellular factor would correlate with the infection rate of the total CD4 population, this correlation would result in a slope of 1 if the infected subset is just a subsample of the total infected pool. This appeared to be the case for memory cells, but not for naive cells. The slope of 3.6 in naive cells suggests that for every increase in the infection rate of the naive compartment, there was a corresponding approximately 4-fold increase in the infection rate of the total CD4 compartment. Although these mathematical predictions do not define causality, these results are indicative of an outsized contribution of naive cells to the HIV reservoir.

Overall, these findings suggest that naive cells contributed minimally to the reservoir of ECs, yet they contributed significantly to the reservoir in CPs especially as reservoir size increases.

### Critical role for naive T cells in HIV persistence in CPs.

In recent years, NFL sequencing has increased our understanding of the proviral landscape in individuals with HIV infection (18–22). To genetically characterize the composition of the HIV reservoir in sorted cellular subsets of individuals with different levels of HIV control, we sequenced 5843 proviruses from the enrolled 10 participants (Table 2; ref. 10). Given the small reservoir size of ECs, we utilized more than 1 billion peripheral blood mononuclear cells (PBMCs) from each individual to obtain enough cells for downstream applications (Table 2). Supplemental Table 2 shows the levels of HIV DNA and the contribution of each cellular subset to total CD4^+^ T cells.

**Figure 2:**
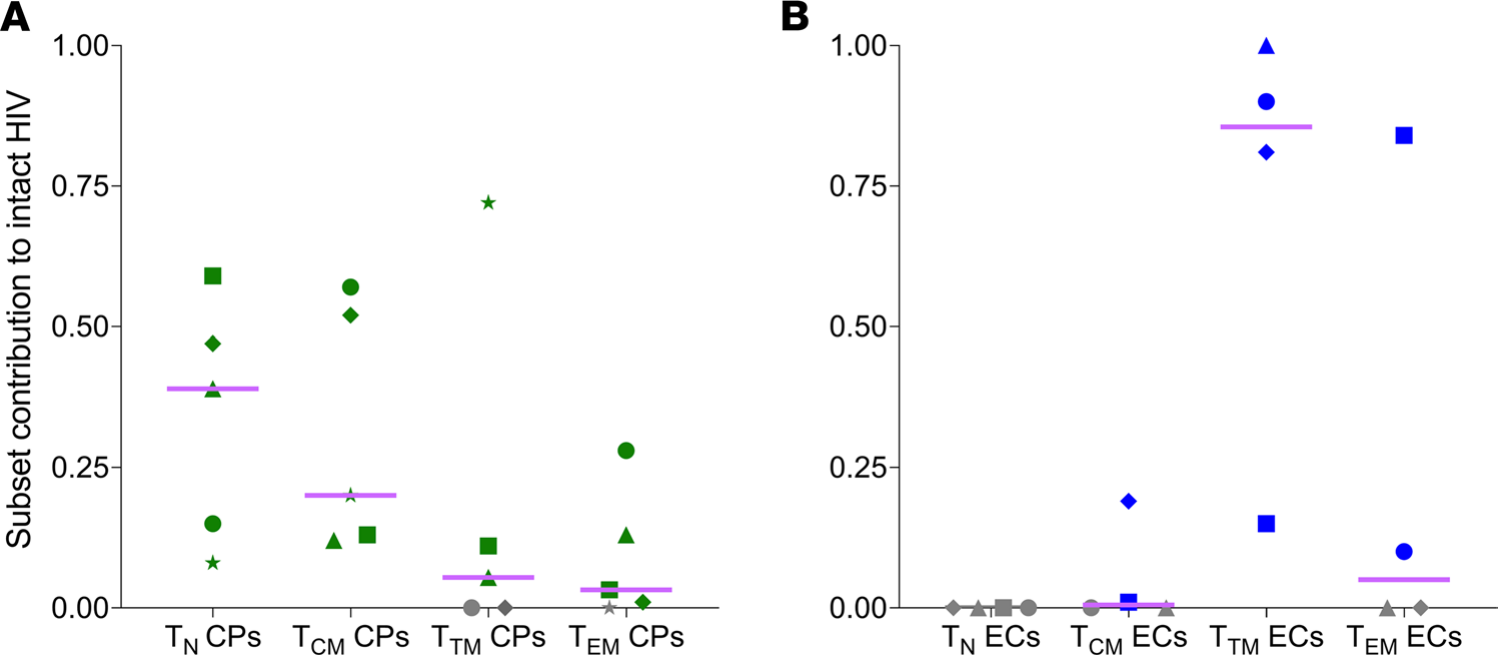
The intact reservoir is substantial in naive T cells of CPs but largely contained by memory cells in ECs. (**A**) Contribution of T cell subsets to the intact reservoir of CPs 2–3 years after ART initiation (*n =* 5). Naive cells represented a major contributor to the intact reservoir, with a median contribution of 39% (IQR, 12%–53%), followed by Tcm cells (20% [IQR, 13%–55%]) >Ttm cells (6% [IQR, 0%–42%]) >Tem cells (3% [IQR, 0.005%–21%]). (**B**) Contribution of T cell subsets to the intact reservoir of ECs (*n =* 4). Naive T cells did not contain any intact HIV sequence. Ttm cells were the major contributor to intact HIV in ECs (86% [IQR, 32%–98%]). No intact HIV sequences were found in Tcm cells and Tem cells of 2 ECs (gray symbols). EC1 was excluded because we did not retrieve any intact HIV sequence. The contribution of each subset to the intact reservoir was calculated using the following formula: [(levels of HIV DNA within subset) × (% intact HIV within subset) × (subset/μL/total CD4/μL)]. Lines represent median values. ART, antiretroviral therapy; CPs, chronic progressors; ECs, elite controllers; naive T cells, naive CD4^+^ T cells; Tcm, central memory CD4^+^ T cells; Ttm, transitional memory CD4^+^ T cells; Tem, effector memory CD4^+^ T cells.

The contribution of each cellular subset to the intact reservoir is shown in Figure 2 and Table 4. Supplemental Figure 1 shows the overall proportion of intact HIV in CD4^+^ T cells. We did not identify any intact proviral sequence in the naive cells of ECs (Figure 2B), which is consistent with the extremely low levels of naive infection of these participants (Table 4). In CPs, naive cells were a major contributor to the intact reservoir (Figure 2A). Early after ART initiation, naive cells contribute between 8% and 59% of the intact reservoir, but they contributed less intact HIV in CPs with smaller reservoirs and better immunological control (CP1 and CP2; Table 1). Four to eight years after ART initiation, naive contribution to intact HIV ranged between 20% and 34% (data not shown). The naive contribution was higher still when we minimized the effect of clonal expansion by removing repeated sequences (data not shown), consistent with a previous study from our group (10).

### The naive reservoir may be more protected due to lower HIV RNA expression levels in CPs.

**Figure 3:**
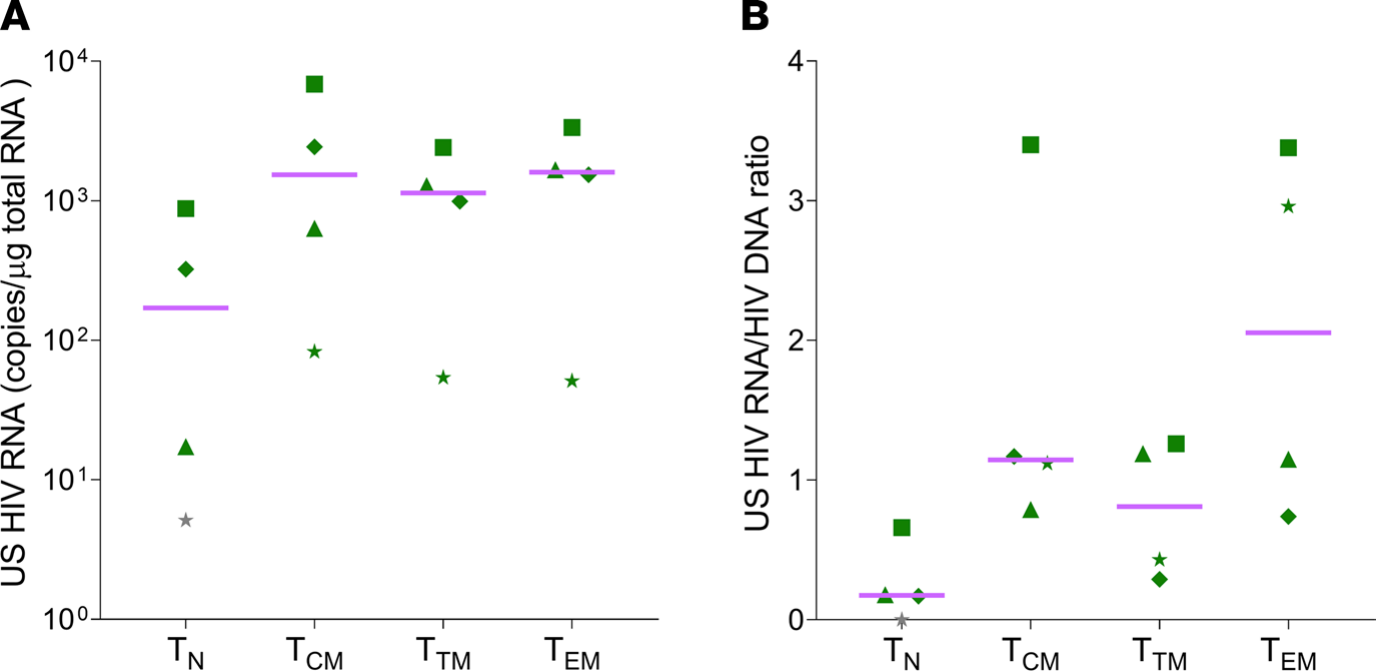
HIV RNA levels in naive and memory cells of CPs. (**A**) Levels of US HIV RNA were significantly lower in naive cells (median value 171 [IQR, 8.2–739] copies/μg total RNA cells) when compared with memory cells (*P =* 0.02 by Friedman’s test) in 4 CPs on ART. The comparison was statistically significant only in Tcm vs. naive T cells (*P =* 0.04), likely due to the small sample size. Levels of US HIV RNA in memory cells were similar across subsets, ranging from 1534 (IQR, 221–5748) to 1136 (IQR, 288–2129) to 1601 (IQR, 422–2933) copies/μg total RNA in Tcm, Ttm, and Tem cells, respectively. (**B**) More importantly, the US HIV RNA/HIV DNA ratio trended toward lower values in naive cells (0.18 [0.04–0.54]) compared with memory cells (1.1 [0.92–2.5]). CP2 was excluded as the levels of HIV RNA were undetectable. With the exception of EC5, none of the ECs had detectable levels of HIV RNA in unfractionated CD4^+^ T cells. Lines represent median values. Groups were compared using the Friedman’s test. The Dunn’s test was used to correct for multiple comparisons. The gray symbols depict undetectable values, censored to 50% of the corresponding detection limits. ART, antiretroviral therapy; CP,s chronic progressors; ECs, elite controllers; naive T cells, naive CD4^+^ T cells; Tcm, central memory CD4^+^ T cells; Ttm, transitional memory CD4^+^ T cells; Tem, effector memory CD4^+^ T cells; US, unspliced.

The relatively high frequency of intact HIV DNA in naive T cells suggests a selective advantage. We reasoned that naive cells might be protected from immune clearance relative to memory cells because they express lower levels of HIV RNA, consistent with prior studies (23, 24). We measured the levels of unspliced (US) HIV RNA and the US RNA/DNA ratio in cellular subsets in the CP cohort (Figure 3). The levels of US RNA were overall lower in naive cells compared with memory subsets (*P =* 0.02; Figure 3A), though this difference was statistically significant only for naive T cells versus Tcm cells, likely due to the small sample size (*P =* 0.04). More importantly, the US HIV RNA/DNA ratio tended to be lower in naive cells (Figure 3B). The lower HIV expression in naive cells might provide additional protection from immune and/or viral cytotoxicity (31), leading to a survival advantage for intact proviruses in these cells.

### Proviral diversity steadily decreased with cell differentiation and was largely predicted by naive infection.

The proliferation of cells containing HIV is a major determinant of HIV persistence (25, 26), because clonal expansion of infected cells counterbalances reservoir decay at any moment in the clinical history of individuals with HIV (21).

**Figure 4:**
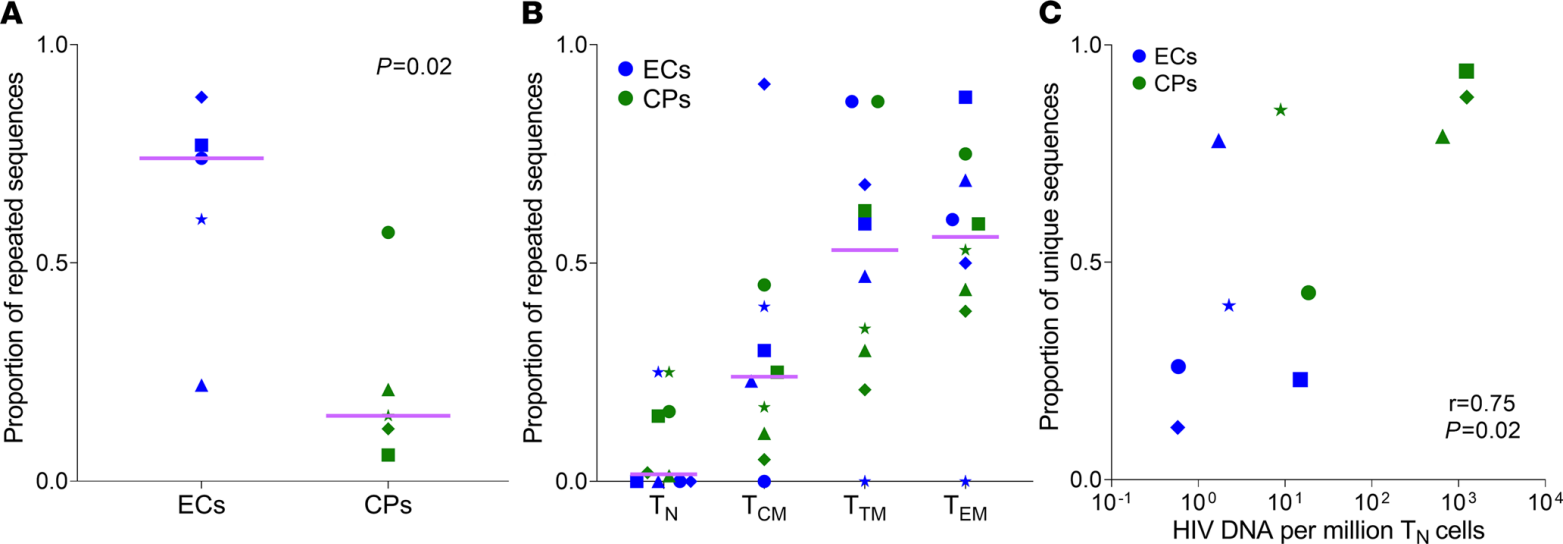
The diversity of the HIV reservoir correlates with the levels of naive infection and decreases as cells become more differentiated. (**A**) The proportion of repeated sequences detected by NFL proviral sequencing was significantly higher in CD4^+^ T cells of ECs compared with CPs (74% [IQR, 41%–83%] vs. 15% [IQR, 9%–39%], *P =* 0.02). (**B**) The fraction of repeated sequences steadily increased from naive T cells (2% [IQR, 0%–18%]) to Tcm (24% [IQR, 10%–41%]) to Ttm (53% [IQR, 28%–73%]) to Tem (56% [IQR, 43%–71%] cells, *P =* 0.002), reaching statistical significance for naive T cells vs. Tem (*P =* 0.006) and naive T cells vs. Ttm (*P =* 0.01). (**C**) The levels of naive infection significantly correlated with the proportion of unique sequences in CD4^+^ T cells (*r* = 0.75, *P =* 0.02). All panels include 5 ECs off ART and 5 CPs whose cells were collected 2–3 years after ART initiation. Lines represent median values. Groups were compared using the Mann-Whitney *U* test in **A** and Friedman’s test in **B** (with Dunn’s correction for multiple comparisons). Levels of HIV DNA are reported as copies/million cells. The correlation in **C** was calculated using the Spearman’s coefficient correlation. ART, antiretroviral therapy; CPs, chronic progressors; ECs, elite controllers; NFL, near-full-length; naive T cells, naive CD4^+^ T cells; Tcm, central memory CD4^+^ T cells; Ttm, transitional memory CD4^+^ T cells; Tem, effector memory CD4^+^ T cells.

We wanted to compare the reservoir diversity in ECs versus CPs. To do this, we measured the fraction of repeated sequences in the 2 cohorts. We defined these identical proviral sequences that were detected more than once by NFL proviral sequencing as “proviral clones.” The proportion of repeated sequences was significantly higher in ECs versus CPs (*P =* 0.02; Figure 4A). The fraction of repeated sequences in cellular subsets steadily increased from less mature to more mature T cells (naive T cells < Tcm < Ttm < Tem; Figure 4B; *P =* 0.002; ref. 10). Moreover, the strongest correlation occurred between the proportion of unique sequences and the levels of HIV DNA in naive T cells (Figure 4C; *P =* 0.02; Supplemental Table 3). We obtained similar results using another metric of diversity, the Simpson Index without replacement (*P =* 0.03). Thus, our results suggest that a smaller naive reservoir results in less overall reservoir diversity.

### The proviral landscape of ECs was dominated by few proviral clones.

**Figure 5:**
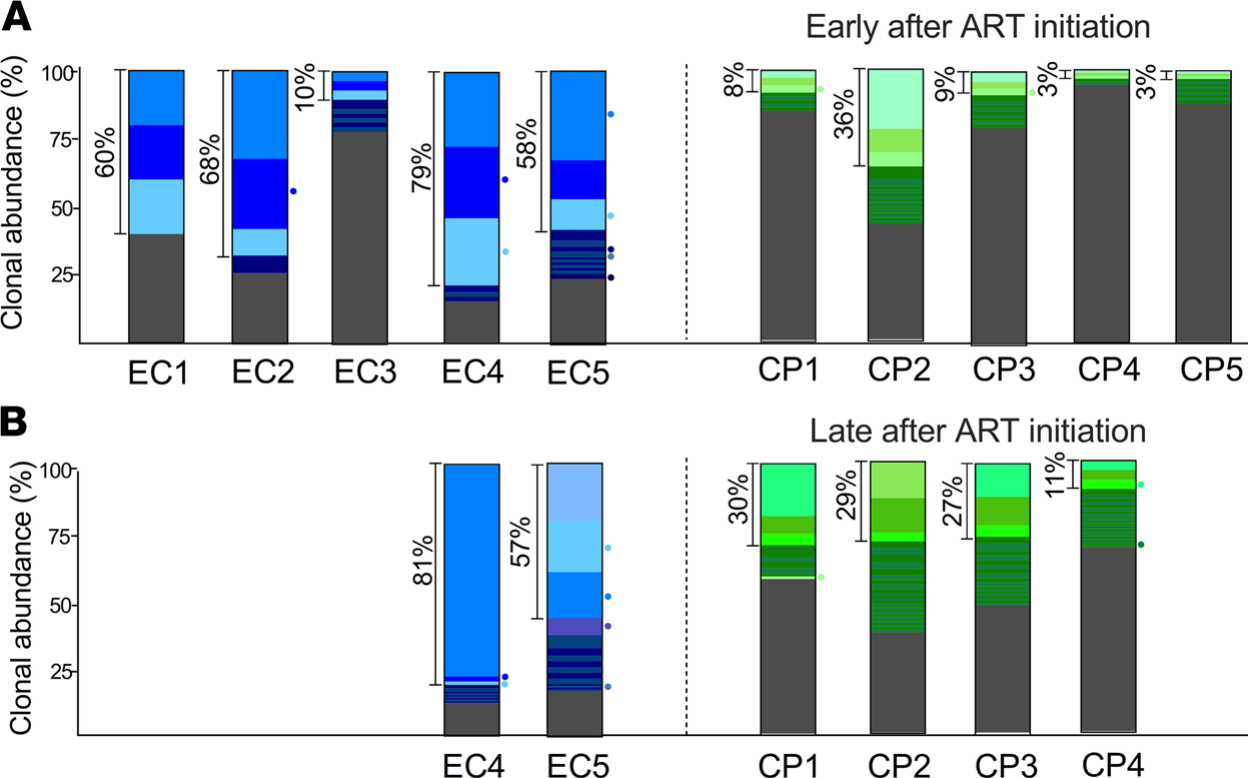
Frequency of dominant proviral clones is higher in ECs. (**A**) The majority of the proviral sequences retrieved from CD4^+^ T cells belonged to 3 largely expanded proviral clones (58%–79%) for 4 of 5 ECs. This fraction was lower in CPs early after ART initiation (2–3 years), representing only 3%–9% of the reservoir for 4 of 5 CPs. (**B**) The fraction and composition of repeated sequences were quite stable over time in ECs but changed in CPs on ART. The fraction of predominant clones in ECs was stable in these 2 participants compared with the early time point (**A**), whereas the proportion of sequences contributed by the highly expanded clones increased for CPs with time on ART (11%–30%). For CPs, the predominant clones at the late time point were different from the predominant clones identified at the first time point, except for 1 proviral clone in CP2, which persisted at both time points. For ECs, the predominant clones were the same at both time points, with the exception of 1 proviral clone in EC5, though the relative percent contribution of each clone changed over time. The gray fraction represents unique sequences, whereas the shades of blue (for ECs) or green (for CPs) identify distinct proviral clones. The circles on the right side of each bar identify intact proviral clones. The same color was used when the intact clone persisted at both time points. The percentages represent the fraction of total sequences in CD4^+^ T cells/PBMCs made up by the 3 predominant proviral clones. Proviral clones were identified as identical repeated sequences using a larger database of proviral sequences from multiple time points for each participant as summarized in Table 5. Notably, we subsampled our database for CP3 and CP4 to include only PBMCs from 4 time points (21) to keep the number of sequences among CPs similar. Overall, we sampled fewer sequences among ECs due to lower levels of proviruses; nonetheless, we found more clones in ECs compared with CPs. ART, antiretroviral therapy; CPs, chronic progressors; ECs, elite controllers; NFL, near-full-length; PBMCs, peripheral blood mononuclear cells.

Because a recent study showed that the EC proviral landscape was more clonal (27), we wanted to know if our cohort followed a similar pattern. As a metric of clonality, we measured the proportion of sequences represented by the 3 most abundant proviral clones in ECs versus CPs. The size of the proviral database and the number of repeated sequences for each individual are shown in Table 5. We found that in ECs, the 3 most frequent clones represented the majority of their reservoir, whereas in CPs this fraction was below 10% early after ART initiation (Figure 5A). Notably, CP2 did not follow the same pattern, as the most abundant clones represented 36% of the total sequences. This might have been due to the expansion of 1 or few large T cell clones in response to recent infection with syphilis. In addition, 1 EC (EC3) had a lower fraction of repeated sequences compared with the other ECs. EC3 lacks the protective HLA alleles that are generally associated with the EC status (such as B27 and B57; ref. 28; Table 1), but this observation will need to be studied in a larger cohort to substantiate this association.

To assess the effect of ART on the clonal composition over time, we performed the same analysis at a later time point on unfractionated CD4^+^ T cells of 2 ECs and 4 CPs (Figure 5B). We found that the frequency and composition of proviral clones were quite stable in ECs, whereas the fraction of sequences represented by the most abundant clones increased in CPs (Figure 5B), suggesting that over time CPs appeared more similar to ECs. Interestingly, the 3 predominant clones identified in CPs at a late time point were different than those identified at an earlier time point, consistent with a more dynamic reservoir in CPs (29). For ECs, the predominant clones did not change over time, with the exception of 1 proviral clone in EC5, though the relative contribution of each clone was different between time points. Taken together, these findings suggest that the reservoir might be relatively more stable in ECs compared with CPs, though not static in absolute terms.

### The predominant proviral clones were stable in ECs while highly dynamic in CPs on ART.

**Figure 6:**
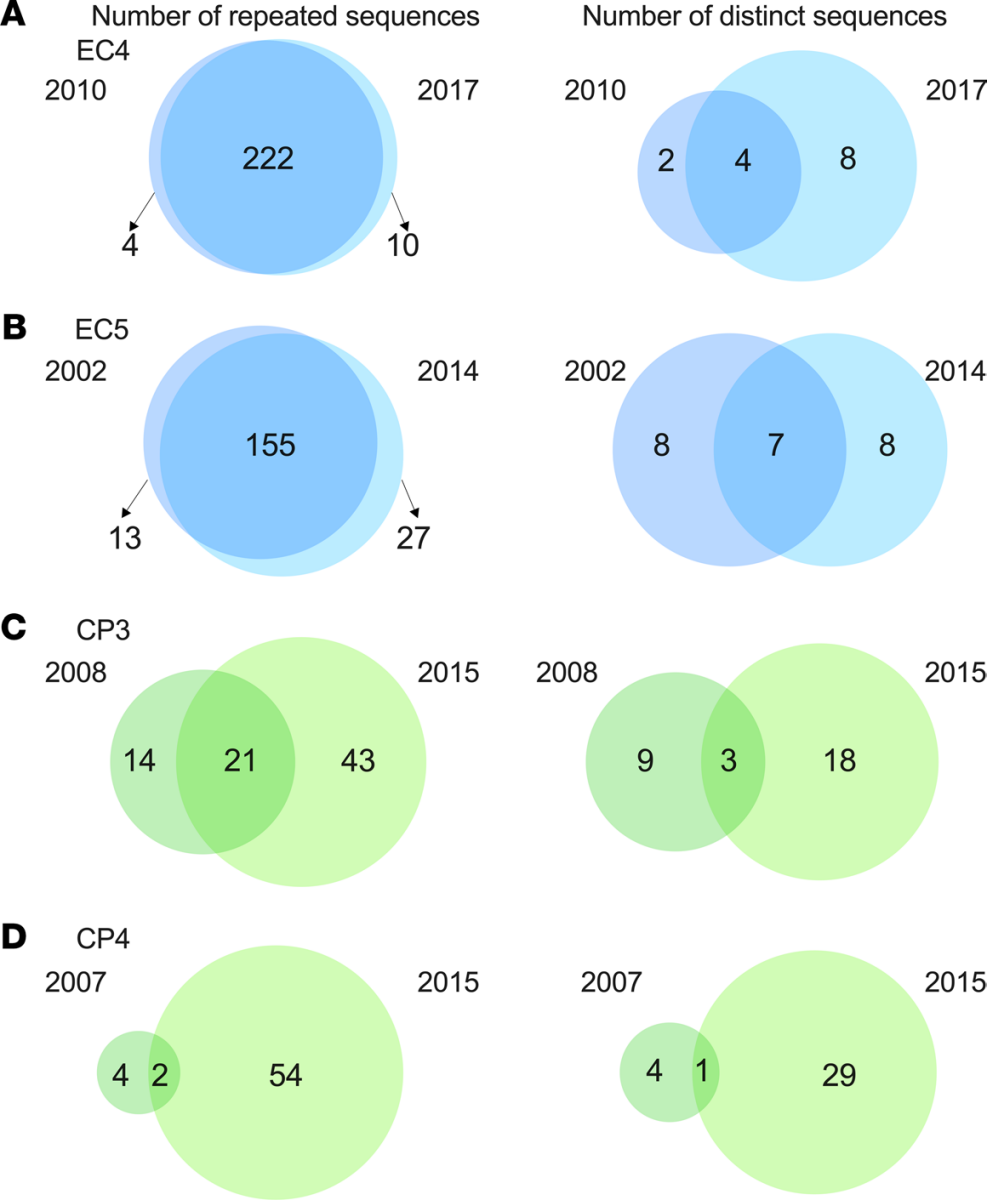
Analysis of clonal overlap over time reveals the oligoclonal nature of the EC reservoir compared with CPs. The proviral clones from Figure 5 were analyzed from EC4, EC5, CP3, and CP4. Proviral clones were defined as any sequence that occurred more than once within the same participant. We identified clonal sequences using the entire database of each individual. The database size was as follows: 545 sequences for EC4, 902 (EC5), 612 (CP3), and 916 (CP4). The centers of the Venn diagrams contain proviral clones present at 2 time points 7 to 12 years apart from each other, whereas the nonoverlapping regions represent proviral clones detected only at 1 time point, though present more than once in the individual’s database. The participants included are EC4 (2010 and 2017), EC5 (2002 and 2014), CP3 (2008 and 2015), and CP4 (2007 and 2015). The left side of the Venn diagram represents the number of proviral sequences that were detected from CD4^+^ T cells/PBMCs at 1 or both time points. The right side of the Venn diagram limits the number of proviruses to those that are distinct. Overall, ECs showed a much larger overlap between time points compared with CPs. CPs, chronic progressors; ECs, elite controllers; NFL, near-full-length; PBMCs, peripheral blood mononuclear cells.

Our results suggest that proviral clones were more stable in ECs compared with CPs. To capture the changes among dominant clones in CPs and ECs over time, we represented proviral clones using Venn diagrams. We utilized sequences from 2 ECs (EC4 and EC5) and 2 representative CPs (CP3 and CP4), for which we had performed NFL proviral sequencing at 2 time points 7 to 12 years apart from each other. Table 5 shows the number of total and overlapping sequences for each individual as well as the size of the database utilized to identify repeated sequences. For each participant, the Venn diagram on the left shows the number of repeated sequences that were detected at either time point or at both time points. As an example, for EC4, 222 of 236 repeated sequences were shared between 2010 and 2017, whereas only 14 sequences were detected in either 2010 or 2017 (Figure 6A, left). The right side of the Venn diagram shows the number of distinct proviral sequences that were detected at either time point or both time points. For EC4, 94% of the repeated sequences were made up of just 4 distinct proviral sequences (Figure 6A, right). A similar pattern was found in EC5 (Figure 6B). In contrast, CPs had a much more diverse reservoir, with less overlap between the 2 time points (as low as 3% for CP4; Figure 6D). In CPs, only a small fraction of clones was detected at both time points. As an example, for CP3, we detected 9 distinct proviral clonal sequences in 2008 and 18 in 2015, whereas there were only 3 of such sequences at both time points. Overall, our data suggest that the HIV reservoir was more dynamic in CPs than in ECs.

## Discussion

In this study, we uncover evidence that naive cells may have played an important role in HIV persistence. Our most striking finding is that naive infection predicted reservoir size and diversity. Intriguingly, we also find that despite being the smallest HIV reservoir, infected naive cells contained the most unique sequences of all T cell subsets (Figure 4B; ref. 10). The simplest explanation is that the direct infection of naive T cells must occur in vivo, challenging the dogma of naive infection solely by reversion (30). Our work also provides additional evidence that the naive reservoir may replenish the memory reservoir (10, 31, 32). Specifically, we find a steady increase in the fraction of repeated sequences as cells become more differentiated (10, 31, 32). Finally, we find ECs have minimal naive infection and a more oligoclonal reservoir bolstering a role for naive repopulation in reservoir diversity.

Extensive literature supports the idea that the reservoir forms when an activated T cell becomes infected before it reverts to a memory T cell (30, 33). Although this literature demonstrates that activated T cells are more easily infected, it neglects to consider the selective advantage that naive T cells provide to a provirus. Proviruses within naive T cells are more diverse than Tcm > Ttm > Tem (Figure 4B; ref. 10). This descending diversity implies direct infection of naive cells contributed to reservoir formation. If every infected naive cell formed by reversion of proliferating memory cells, then we would expect most naive sequences to be clonal with most naive proviral sequences also present within the memory cells; on the contrary, we find that naive T cells have the largest number of unique sequences, suggesting that these cells were directly infected.

Infected naive T cells have unique properties that make them a formidable reservoir. This includes their long intermitotic half-life (31, 34), their inherent resistance to cytotoxic T lymphocyte (CTL) clearance (35), and their resistance to expression of HIV at the level of RNA (Figure 3; refs. 23, 24, 31) and protein (36). We provide additional evidence that proviruses within naive T cells are shielded from selection pressures, because we find intact proviruses are relatively preserved over defective proviruses in vivo (9, 10). The level of naive infection in vivo is all the more impressive given their relatively resistance to infection in vitro (37). Our findings are supported by a recent study (31) measuring the fractional replacement rate of different cellular subsets and clonal turnover. The authors drew similar conclusions using complimentary approaches that naive T cells provide a pool of infected cells to repopulate the reservoir. Overall, our study suggests that direct infection of naive T cells in vivo may have played a larger role in reservoir formation and maintenance than previously recognized.

Although studies have shown varying levels of naive reservoir in chronic infection (4, 8, 22, 38–41), our work provides a rationale for this variation and shows a continuum of naive infection related to the size of the reservoir. We deliberately chose to study a continuum of infection rather than compare 2 homogenous groups. Thus, we enrolled individuals with a spectrum of reservoir sizes and levels of immune control. Although studying 2 uniform populations of HIV-infected individuals may have resulted in stronger levels of significance when comparing the level of clonality and naive infection, our approach provided additional insights revealing a continuum of reservoir size, and coreceptor tropism. We clearly found that those individuals with larger and predominant CXCR4-tropic reservoirs had more naive infection, consistent with a prior study (42), and more diverse reservoirs. This finding is reasonable because CXCR4-tropic viruses can directly infect naive T cells. Thus, these CXCR4-tropic viruses provide a potential explanation for the naive-dominant reservoir with high diversity observed in some individuals.

Our work raises several questions related to the infection of naive T cells. First and foremost, CCR5-tropic proviruses appear to be present in naive cells in vivo (10, 42), despite their in vitro resistance to infection with CCR5-tropic HIV (37). Perhaps, this barrier is overcome by transient upregulation of CCR5, leading to increased susceptibility after interaction with antigen-presenting cells (30, 43). This may facilitate naive infection in vivo and may explain this dichotomy. Alternately, viruses classified as CCR5-tropic by computer algorithms might be dual-tropic (44–46). The fact that extensive viral evolution correlates with the highest levels of naive infection argues that CXCR4-tropic and dual-tropic HIV viruses may be responsible for the majority of naive infection in our CPs. Finally, reversion from a memory to a naive phenotype could certainly provide a mechanism for naive infection, especially by CCR5-tropic HIV (30). Similarly, the minimal naive infection in ECs may be related to the minimal viral evolution in this cohort as well as lower levels of CCR5 reported in ECs (47, 48). Because ECs have predominant CCR5 tropism and lower peak viral loads than CPs (49), we postulate that the negligible levels of naive infection in ECs derive from absence of infection rather than preferential clearance.

The character of the HIV proviral landscape is dramatically different in ECs compared with CPs. We found the EC reservoir to be oligoclonal, consistent with prior studies (27, 50, 51). Intriguingly, these proviral clones appear to be relatively stable over time (Figure 5 and Figure 6). A potential explanation is that the majority of the EC reservoir is cleared in the early stages after infection, due to potent and broad CTL responses (52, 53). The residual proviral burden might derive from proviruses that are integrated in nongenic or pseudogenic regions of the genome (27) and have a lower potential for HIV expression, as recently proposed (27). These “silent” proviruses might experience less immune pressure. We speculate that when the naive reservoir is minimal, there is less potential for naive cells to repopulate the memory reservoir and this might contribute to an oligoclonal reservoir, as seen in ECs. It is interesting that CPs begin to resemble ECs after several years on ART (Figure 5; ref. 27) because they become more oligoclonal in character, presumably due to immune selection, albeit less effective than in elite controllers.

Given the extremely small reservoir size of ECs, we opted to exclude Tscm and CD45RA_dim_ cells (10, 54) and did not identify integration sites. We predict the oligoclonal proviruses of ECs would be enriched in “gene deserts,” as previously shown (27). We also used limiting dilution PCR to estimate reservoir size in individuals with small reservoirs, which has a higher coefficient of variation than quantitative PCR (qPCR). Another limitation of this study is that we only included ECs off ART. Thus, we could not match ECs to CPs based on their time on ART, nor did we match ECs to CPs based on their level of diversity or AUC of viremia because this is difficult to control for (15–17, 55, 56). Related to the very small reservoir size of ECs, there is limited data on the EC proviral landscape, especially on ART (27, 57–59). Finally, although our study included an unprecedented number of proviral sequences, our findings need to be confirmed in larger cohorts.

In summary, we provide the first evidence to our knowledge of direct infection of naive T cells in vivo. Unique properties of naive T cells make them a formidable reservoir. These include their potential to avoid immune clearance and their ability to repopulate the memory reservoir through cell differentiation. These features underlie their outsized contribution to reservoir size and diversity, supported by our observation that ECs had less naive infection with a minuscule and oligoclonal reservoir. Although larger studies are needed to explore the prognostic value of naive infection, naive cells should represent a focal point of future research aiming at perturbing the HIV reservoir.

## Methods

### Participants and samples.

Cells from 5 ECs and 5 CPs were collected by apheresis (Table 1, Table 2, and Supplemental Table 1). Cells from ECs were provided in-house. Table 1, Table 2, and Supplemental Table 1 provide additional information on the clinical characteristics and collection time points of the 2 cohorts. Every participant had undetectable viral load (defined as HIV RNA <50 copies/mL blood) at the time of apheresis. ECs were off ART but had maintained undetectable viral loads for the vast majority of longitudinal collections since diagnosis. The following exceptions were observed: EC4 had 1 viral blip in 2011 (53 copies/mL blood; EC2 had 2 blips (in 2001, 68 copies/mL blood, and in 2003, 125 copies/mL blood); and EC5 had 1 isolated viral blip in 1998 (1787 copies/mL blood), preceded and followed by continuously undetectable viral loads from 1995 to 2014 (last collection time point for this study). CPs had been on ART for 2 to 3 years at the time of the first apheresis collection and had undetectable viremia for at least 1 year at enrollment. The following viral blips were observed during the study: CP2 had 1 isolated blip in 2016 (138 copies/mL blood) and CP4 had 2 blips (123 and 99 copies/mL blood in 2006 and 2008, respectively).

### Cell sorting.

CD3^+^CD8^–^ T lymphocytes were negatively selected from PBMCs and sorted on the BD FACS Aria III sorter into the following subsets: CD95^–^ naive T cells (CD45RA^+^CCR7^+^CD27^+^CD95^–^), Tcm (CD45RA^–^CCR7^+^CD27^+^), Ttm (CD45RA^–^CCR7^–^CD27^+^), and Tem cells (CD45RA^–^CCR7^–^CD27^–^), as described in ref. 10, using BV421 anti-CD45RA (clone 5H9, BD Biosciences), PE-Cy7 anti-CD27 (clone 0323, Invitrogen), APC anti-CD95 (clone DX2, BD Biosciences), and BB700 anti-CCR7 (clone 3D12, BD Biosciences). FlowJo v10.6 software was used for analysis.

### HIV DNA quantification and NFL proviral sequencing.

Genomic DNA was purified using the Gentra Puregene Kit (QIAGEN), and HIV DNA was measured by HIV long terminal region qPCR (21) for CP2, CP3, CP4, CP5, and EC5. For the remaining individuals, due to cell number restrictions (and overall smaller reservoir size), we used limiting dilution PCR to estimate the levels of HIV DNA (60–62). We validated the limiting dilution PCR methodology by determining the efficiency of PCR after amplifying proviruses from well-characterized CPs at limiting dilution. We then calculated a correction factor by dividing the number of HIV copies determined by qPCR by the number of copies predicted by Poisson distribution. The final calculation was –4.5*ln (fraction of empty wells). Finally, in a validation data set, we demonstrated the LD measures were not statistically different from the qPCR measures.

Proviral sequences were obtained by long-range PCR amplification at limiting dilution followed by sequencing on an Illumina MiniSeq. Our bioinformatics pipeline is described in ref. 21. Briefly, proviral sequences were de novo assembled and aligned to HXB2. Intact proviruses were defined according to stringent criteria (21). We used the Multiple Alignment with Fast Fourier Transformation to identify proviral clones, defined as sequences with 100% identity (63).

### US HIV RNA quantification.

The levels of US HIV RNA were measured by qPCR using an assay that recognizes the HIV packaging signal (64) and normalized to the total cellular RNA input as measured by 18S ribosomal RNA (65). We did not detect any HIV RNA in unfractionated CD4^+^ T cells of ECs, consistent with a prior study (66), except for EC5 who had an unusually large reservoir. For CPs, due to limited cell numbers, we measured the levels of US HIV RNA only in samples collected from the late time point after ART initiation.

### Coreceptor tropism.

Proviral coreceptors were classified by analyzing the V3 region of the HIV envelope protein gp120 using Geno2Pheno (67), with a 10% false positive rate.

### Statistics.

Nonparametric tests were used for the analysis, including the Mann-Whitney *U* test for unpaired samples and the Friedman’s test for paired samples (with Dunn’s correction for multiple comparisons). Correlations were calculated using the Spearman’s correlation test. The infected naive/memory ratios were computed using a modified Fisher’s exact test adjusted to correct for uneven sampling of subsets. Monte-Carlo methods were used to resample. The infected naive/memory ratios were calculated from discrete counts of total cells analyzed and total number of infected cells found. This provided data as a total (integer) number of positives compared with a total (integer) number of negatives for 2 test conditions (EC or CP). For each cellular subset from each individual, the corresponding beta distribution for the positive frequency was calculated. The subset beta distributions were then combined into a posterior distribution by resampling according to their relative contributions to total memory. R, Prism GraphPad, and Excel software were used for statistical analyses. *P* values of less than 0.05 were considered significant.

### Study approval.

The study was approved by the IRBs at the NIH and at the University of Pennsylvania. CPs (and EC4 at the latest time point) were enrolled according to the protocol 704904 and cells from ECs were obtained according to NIH protocol with IRB approval. Participants provided written informed consent before inclusion in the study.

## Author contributions

MRP and UO designed the study. MRP performed the sorting, qPCR, and sequencing experiments. AOP performed the HIV RNA measures. MRP, SW, and UO analyzed the experimental results. SM contributed the EC samples. RZ provided statistical support for data analysis. MRP and UO wrote the paper. All the authors approved the final version of the manuscript.

## Supplementary Material

Supplemental data

## Figures and Tables

**Table 1:**
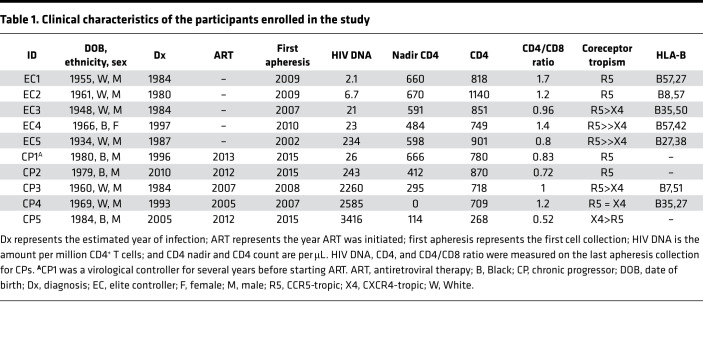
Clinical characteristics of the participants enrolled in the study

**Table 2:**
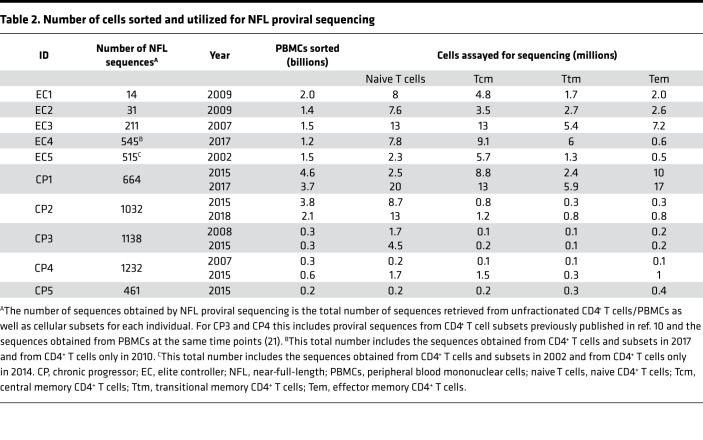
Number of cells sorted and utilized for NFL proviral sequencing

**Table 3:**
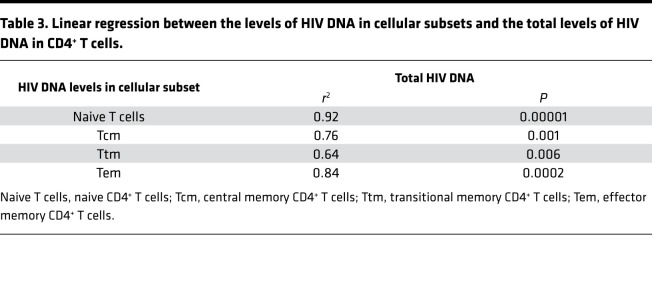
Linear regression between the levels of HIV DNA in cellular subsets and the total levels of HIV DNA in CD4^+^ T cells.

**Table 4:**
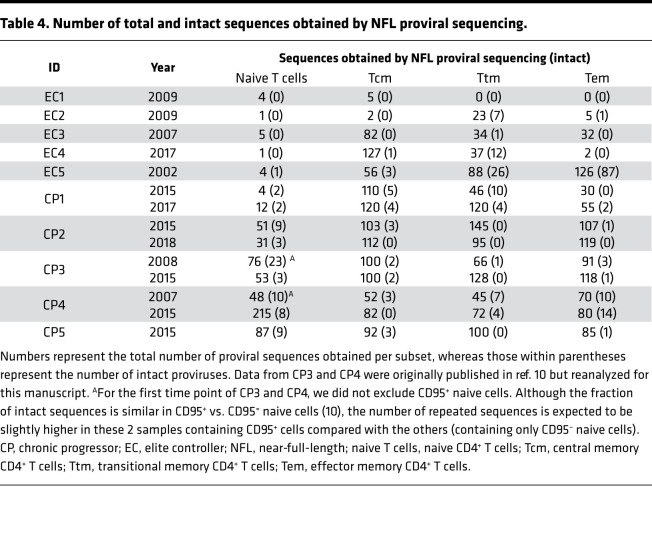
Number of total and intact sequences obtained by NFL proviral sequencing.

**Table 5:**
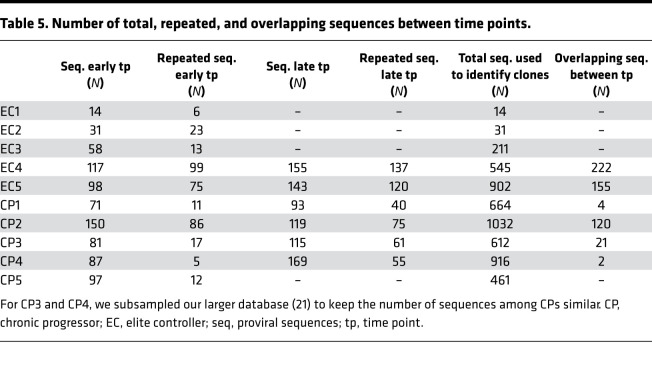
Number of total, repeated, and overlapping sequences between time points.

## Version 1 07/06/2021

In-Press Preview

## Version 2 08/23/2021

Electronic publication
