# Supplementary material for: Naive infection predicts reservoir diversity and is a formidable hurdle to HIV eradication
Source: JCI Insight. 2021 Aug 23;6(16):e150794. doi: 10.1172/jci.insight.150794 (PMC8409977; doi:10.1172/jci.insight.150794)
Supplement: Supplemental data [file jciinsight-6-150794-s077.pdf]

Naive infection predicts reservoir diversity and is a formidable hurdle to HIV eradication

Marilia Rita Pinzone[1], Sam Weissman[1], Alexander O Pasternak[2], Ryan Zurakowski[3], Stephen Migueles[4], Una O'Doherty

Supplementary data

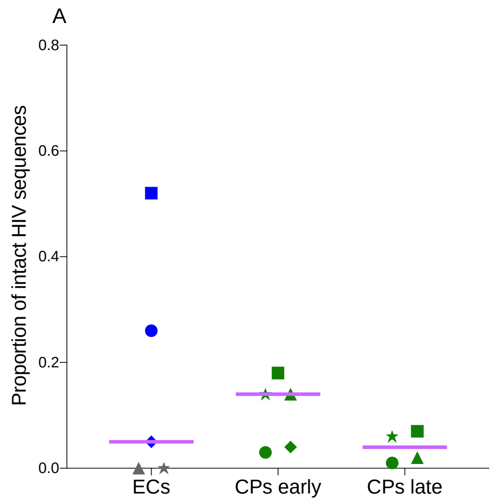

Figure S1. Proportion of intact HIV sequences in ECs and CPs. In the EC group, we did not detect any intact HIV in CD4 T cells from two participants (grey symbols). In the remaining three ECs, the fraction of intact HIV greatly varied ranging between 5 and 52%. For one EC (blue square), about half of the reservoir was represented by intact HIV sequences. The vast majority of these intact sequences were repeated sequences and belonged to two largely expanded clones. These findings are consistent with clonal expansion of infected cells containing intact HIV. In fact, given the high error rate of HIV reverse transcriptase, we would not expect to retrieve the same identical sequence if these results were due to ongoing replication. In CPs, the fraction of intact HIV in CD4 T cells ranged between 3 and 18% at the early timepoint after ART initiation (N=5) and between 1 and 7% at the late timepoint (N=4), consistent with decay of intact HIV over time on ART.

Abbreviations: ART: antiretroviral therapy; CP: chronic progressor; EC: elite controller

Table S1. Additional virological and immunological parameters for the chronic progressors studied at two time points

ID	Apheresis collection (year)	ART regimen	HIV RNA	CD4	CD4/CD8 ratio
CP1	2015	ABC DTG 3TC	<20	1348	0.96
	2017	DTG RPV	<20	971	0.90
CP2	2015	TDF/FTC RPV	<20	871	0.65
	2018	TAF/FTC/EVG/COBI	<20	870	0.72
CP3	2008	ATV/r TDF/FTC	<50	617	---
	2015	ATV/r TDF/FTC	<20	718	1.0
CP4	2007	ATV/r 3TC D4T	<50	629	---
	2015	ATV/r 3TC RAL	<40	768	1.2
CP5	2015	ATV/r TDF/FTC ABC	<20	268	0.52

Abbreviations: ABC: abacavir; ART: antiretroviral therapy; ATV: atazanavir; CP: chronic progressor; COBI: cobicistat; D4T: stavudine; DTG: dolutegravir; EVG: elvitegravir; FTC: emtricitabine; r: ritonavir; RAL: raltegravir; RPV: rilpivirine; TAF: tenofovir alafenamide; TDF: tenofovir disoproxil fumarate; 3TC: lamivudine

CD4 counts are presented as cells/μl, HIV RNA as copies/ml blood

Table S2. Cellular subset contribution to total CD4 T cells and estimates of HIV DNA levels in each subset

ID	Year	Subset contribution to total CD4 (%)				HIV DNA (copies per million cells)			
		T_N	T_{CM}	T_{TM}	T_{EM}	T_N	T_{CM}	T_{TM}	T_{EM}
EC1	2009	37	22	6.7	7.4	2.3	12	<2	<2
EC2	2009	33	16	12	11	0.59	3.9	52	11
EC3	2007	23	29	5.2	9.1	1.7	50	46	32
EC4	2017	18	45	9.5	3.4	0.58	77	35	14
EC5	2002	7.0	16	12	10	15	106	351	1001
CP1	2015	8.1	21	11	30	8.9	95	145	22
CP1	2017	24	21	7.0	20	6.9	73	144	35
CP2	2015	24	19	11	13	19	781	1725	1175
CP2	2018	48	21	3.9	5.7	12	566	826	547
CP3	2008	21	14	7.8	12	655	4581	4915	3694
CP3	2015	23	20	6.8	11	468	2641	4383	3446
CP4	2007	34	9.4	4.0	1.0	1236	3719	2601	3316
CP4	2015	44	16	4.5	1.7	904	2048	1446	740
CP5	2015	31	21	4.8	5.5	1242	6473	2263	2096

The sum of the percentage contributed by each subset is less than 100% because we excluded CD45RA*dim* and T_{EMRA} cells as well as cells that were near the boundary of the sorting gates

Abbreviations: CP: chronic progressor; EC: elite controller; T_N: Naive CD4 T cells; T_{CM}: Central memory CD4 T cells; T_{TM}: Transitional memory CD4 T cells; T_{EM}: Effector memory CD4 T cells; T_{EMRA}: terminally differentiated effector memory T cells

Table S3. Correlation between the levels of HIV DNA in cellular subsets and the fraction of unique sequences

Cellular subset	Unique proviral sequences	
	r	p
T_N	0.75	0.02
T_{CM}	0.61	0.07
T_{TM}	0.64	0.06
T_{EM}	0.62	0.06

Abbreviations: T_N: Naive CD4 T cells; T_{CM}: Central memory CD4 T cells; T_{TM}: Transitional memory CD4 T cells; T_{EM}: Effector memory CD4 T cells
